# Biocompatible Polymer for Self-Humidification

**DOI:** 10.3390/polym15204101

**Published:** 2023-10-16

**Authors:** Ahmed M. Al-Jumaily, Sandra Grau-Bartual, Nimesha T. Weerasinghe

**Affiliations:** AUT—Institute of Biomedical Technologies, Auckland University of Technology, Auckland 1010, New Zealand

**Keywords:** hydrophilic, hydrophobic, humidification, LCST, CPAP

## Abstract

Lung supportive devices (LSDs) have been extensively utilized in treating patients diagnosed with various respiratory disorders. However, these devices can cause moisture depletion in the upper airway by interfering with the natural lubrication and air conditioning process. To remedy this, current technologies implement heated humidification processes, which are bulky, costly, and nonfriendly. However, it has been demonstrated that in a breath cycle, the amount of water vapor in the exhaled air is of a similar quantity to the amount needed to humidify the inhaled air. This research proposes to trap the moisture from exhaled air and reuse it during inhalation by developing a state-of-the-art hydrophilic/hydrophobic polymer tuned to deliver this purpose. Using the atom transfer radical polymerization (ATRP) method, a substrate was successfully created by incorporating poly (N-isopropyl acrylamide) (PNIPAM) onto cotton. The fabricated material exhibited a water vapor release rate of 24.2 ± 1.054%/min at 32 °C, indicating its ability to humidify the inhaled air effectively. These findings highlight the potential of the developed material as a promising solution for applications requiring rapid moisture recovery.

## 1. Introduction

The human respiratory system plays a vital role in heating and moisturizing inspired gases, as inhaling moist, warm air holds significant value for human well-being. In respiratory dynamics, nasal air conditioning accomplishes rapid warming and humidification of inhaled air upon contact with the nasal mucosa during inhalation. The most efficient exchange of gases occurs when the air entering the lungs maintains a temperature of 37 °C, is saturated with 100% relative humidity, and contains approximately 44 mgH2O/L [1]. However, the use of lung supportive devices (LSD), such as continuous positive airway pressure (CPAP) for patients with lung ailments, interferes with the natural air conditioning and moisture preservation process, leading to severe upper airway dryness, which jeopardizes the use of these devices. To remedy this, current LSDs incorporate convective humidification processes to provide the required humidification. These processes are bulky, costly, and patient nonfriendly. However, it has been established that the recovered moisture from the human exhalation process possesses a sufficient amount of water vapor that can be used to meet the minimal humidity threshold crucial for avoiding upper airway dryness associated with CPAP therapy [1]. This study presents an optimization strategy to fabricate a biocompatible thermo-responsive material to achieve an optimal balance of water vapor uptake and release kinetics within one exhalation–inhalation breath cycle. Here, we present a novel biocompatible smart material that is designed to absorb moisture from exhaled air and subsequently release it to effectively humidify the inhaled air and address the issue of dryness associated with LSD.

Materials exhibiting hydrophilicity can absorb water molecules; their moisture release can only be achieved through mechanical force. Smart materials, however, can respond to external stimuli, exhibiting changes in their physical or chemical properties. This response can be triggered by various stimuli, including mechanical, electrical, magnetic, or thermal energy. Further, thermo-responsive smart materials, such as poly(N-isopropylacrylamide) (PNIPAM), can switch between hydrophilic and hydrophobic states, releasing water vapor at a specific temperature. It is also eco-friendly and biocompatible for medical devices [1,2,3].

The molecular structure of PNIPAM comprises an amide-functionalized n-alkane backbone that an isopropyl group follows, significantly impacting its stimuli-responsive behavior. The formation of hydrogen bonds between the amide group and water molecules depends on the molecular orientation [4]. Polymer dispersion can occur due to the strong interactions between amide groups and polar solvents like water, which contain atoms with different electronegativities. As a result, the solvated polymer forms an organized structure composed of amide and isopropyl groups. In addition, PNIPAM polymer can precipitate and change into a coiled conformation due to a transition from hydrophilic to hydrophobic under the temperature increment.

The lower critical solution temperature (LCST) ranges between 30 °C and 35 °C, where changes in free energy cause this transition. The PNIPAM structure is predominantly hydrophilic below the LCST and in the presence of water. The amide groups tend to create intermolecular hydrogen bonds with the nearby water molecules. When temperature increases above the LCST of PNIPAM, hydrogen bonds between water molecules and amide groups of PNIPAM tend to break, resulting in conformational changes of the polymer. Consequently, the water molecules linked with the polymer functional side groups are released into the surrounding bulk water [4,5]. The LCST of this thermo-responsive polymer can be altered by changing its microstructure, enabling intended designs. When hydrophilic groups are added to a PNIPAM polymer, the LCST increases, while the addition of hydrophobic groups causes it to decrease. Introducing hydrophilic acrylic acid into the network structure causes the PNIPAM polymer to shrink rapidly. This behavior allows for the synthesis of a PNIPAM polymer with the desired characteristics. Despite this, the PNIPAM polymer must strike a balance between achieving the necessary specifications and ensuring the ease of manufacture if it is to be suitable for practical industrial applications [6]. The smart behavior of PNIPAM can be altered when synthesized on a substrate, such as in stimuli-responsive membranes or tissue engineering applications. Despite the hydrophilic and hydrophobic effects causing solvation and precipitation to remain the same as in the bulk solution polymer, incorporating PNIPAM chains onto a surface can modify the transition process due to interactions with the substrate and dense packing of the brushes [6,7,8,9].

Yang et al. reported that a cotton fabric coated with PNIPAM and with a rough surface can switch between super hydrophilic and super hydrophobic states upon temperature changes [10]. When the temperature is below the LCST, the polymer creates a hydrophilic state by forming hydrogen bonds with water molecules. However, when the temperature is above the LCST, the polymer becomes hydrophobic by forming intramolecular bonds, releasing the absorbed water molecules. The transition temperature was found to be 32 °C. The collapse of PNIPAM polymer chains on a substrate is a gradual process that occurs from the outer region of the chains towards the substrate surface. As the temperature increases, the brushes of the polymer gradually shrink until they collapse onto the substrate and expel any remaining water. This transition is slower than the bulk polymer, and its rate is affected by the polymer′s molecular weight (Mw), thickness, and chain density. However, the impact on the rate of structural transition has yet to be thoroughly investigated [7,8].

Atom transfer radical polymerization (ATRP) is a controlled polymerization reaction that creates well-defined polymers with precise molecular architecture. This method can produce complex structured polymers with a high Mw and low polydispersity index. A homogeneous system, fast initiation rate, and fast exchange between active and inactive species are necessary to maintain control of the reaction and achieve a narrow polydispersity index and molar mass distribution. The main challenge is eliminating the transition metal once the synthesis is complete, which can be addressed by increasing catalyst activity and performing the process in an inert atmosphere [11,12,13,14]. ATRP was used to synthesize PNIPAM, allowing for diverse structures on different substrate materials without expensive laboratory equipment. The technique has industrial applications for producing smart engineered materials.

**Figure 1:**
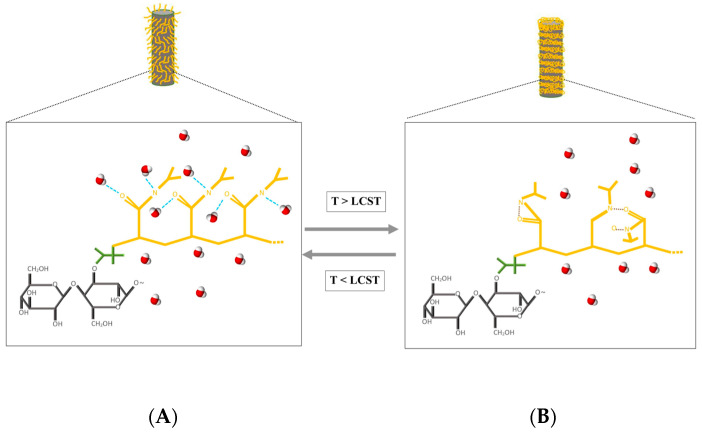
Depiction of a phase change in PNIPAM triggered by temperature on a cotton fiber in a moist environment with water vapor molecules. (**A**) When the environmental temperature is below the LCST, the PNIPAM has a stretched and hydrophilic conformation, oriented perpendicular to the cotton fiber. (**B**) When the environmental temperature exceeds the LCST, the PNIPAM adopts hydrophobic conformation, collapsing around the cotton fiber.

The main objective of this work was to develop a thermo-responsive smart material (PNIPAM) that can rapidly absorb and release water vapor at an LCST within the breath cycle conditions. Specifically, we evaluated various substrate materials and PNIPAM structures and tuned their characteristics to attain the highest possible capacity for rapidly absorbing and releasing water vapor (Figure 1). This is the first reported biomedically applicable PNIPAM-based smart material optimized to absorb and release the optimum water vapor for humidifying inhaled air. It achieves this by recovering moisture from exhaled air and operates within breath cycles lasting 4 to 6 s.

## 2. Materials and Methods

### 2.1. Substrate Materials

The polymer structure′s Mw and dispersity significantly influence the thermal transition and structural behavior of synthesized PNIPAM chains. The substrate material selection is critical to enable the maximum number of active sites for PNIPAM growth, directly impacting the polymer chain dispersity. The substrate must have many alcohol side groups to promote high grafting yield and maximum water intake. The final smart material product must be biodegradable and environmentally friendly.

Two substrate materials, polyvinyl alcohol (PVA) and organic cotton, were selected for the analysis based on the requirements. Due to its water-soluble nature, PVA is a hydrophilic, colorless, and odorless polymer used in various applications such as textiles, papermaking, coatings, and adhesives. It is an atactic material demonstrating crystallinity, and 1,3-diol linkages make up its microstructure. It has a melting point of 200 °C, and over this point, the polymer degrades. PVA is commercially available in a sponge-like macrostructure with heterogeneous porosity that permits the unhindered passage of water vapor molecules [15,16,17,18,19,20,21].

Organic cotton is a natural, soft, and white fibrous substance that grows around the seeds of the cotton plant. Cellulose is the primary component of the cotton structure, forming a long chain of glucose molecules linked by glycoside bonds. Cellulose has a high degree of polymerization and crystallinity due to the chemical reactivity of the three hydroxyl groups (one primary and two secondary) present in each repeating cellobiose unit of cellulose, which keeps the fiber molecules tightly packed in parallel. In addition to being durable, cellulose fibers can withstand temperatures of up to 250 °C before decomposing [22,23]. To ensure sterility and purity, the cotton fabric utilized in this study was bought as medical bandaging.

### 2.2. Chemicals

The following chemicals were used to polymerize the PNIPAM: ethanol (99.5%), methanol (99.9%), acetone (99.8%), dry tetrahydrofuran (99.8%), 4-(dimethylamino) pyridine (DMAP) (99%), triethylamine (TEA) (99%), bromoisobutyrate (BiB) (99%), copper(I)bromide (CuBr) (98%), N-Isopropylacrylamide (NIPAM) (99.5%), pentamethyldiethylenetriamine (PMDETA) (99%), ethyl 2-(bromomethyl) acrylate (EBMA) (98%), dimethylformamide (DMF) (99.8%), deuterated 4-methanol (Methanol-d4) (99.96%), and sodium hydroxide (NaOH) (97%)

### 2.3. Methodology

#### 2.3.1. Linear PNIPAM Synthesis

The procedure involves two modification stages, one for the substrate and the other for the polymerization of the grafted PNIPAM. Modifying the surface of the substrate or immobilizing the ATRP-initiator onto the substrate was achieved by a reaction between one bromine atom of the initiator (known as bromoisobutyrate or BiB) and one alcohol group present on the substrate. A covalent bond between oxygen and bromine links the substrate and the initiator together [6]. The cotton fabric was washed with an aqueous soap-detergent solution under magnetic stirring for 1 h at 100 °C, then rinsed with water, acetone, and ethanol, and then dried under vacuum. This step was repeated three times. The samples were kept at 80 °C under vacuum for 24 h to dry the samples thoroughly. UV light was then used to activate the alcohol groups on the cotton fabric. The cleaned cotton fabric was placed in a flask with tetrahydrofuran (THF) left in an ultrasonic bath and then sealed with a nitrogen balloon to obtain an inert environment. Afterwards, DMAP, TEA, and excess BiB were added to the flask, and the reaction mixture was stirred at 40 °C for 24 h. The resulting samples were rinsed with THF, ethanol, acetone, and water three times and dried under a vacuum.

In the second stage, the monomer, NIPAM (N-isopropylacrylamide), underwent a reaction with the second bromine atom of the ATRP-initiator that was already bonded to the cotton fabric. This stage involved several steps. First, a mixture of methanol and Milli-Q water (30:10 *v*/*v*) was introduced into a round-bottom flask equipped with a magnetic stirrer. Then, the flask was sealed and connected to an active nitrogen gas line, which was placed in an ultrasonic bath for 30 min to create an inert atmosphere. The modified BiB–cotton substrate and a catalytic amount of CuBr and NIPAM were introduced into a Schlenk flask. A methanol/water solution and PMDETA were added to the inert flask, and the entire solution was cooled using liquid nitrogen to achieve complete freezing. The reaction was carried out at room temperature under a continuous nitrogen flow, gently stirring using a magnetic stirrer. Afterwards, the PNIPAM–cotton fabric was subjected to a stepwise washing procedure to eliminate any remaining monomers and the catalyst complex.

The PNIPAM–cotton hermos-responsive material was designed to interact with the human breathing cycle. Therefore, it was crucial to purify the material and remove any remaining precursors before conducting the humidification experiments. The fabricated substrate underwent Soxhlet extraction with methanol under reflux conditions for 1 h, followed by drying at 50 °C under vacuum for 24 h and storage in a 50 °C incubator to ensure purity and dryness.

#### 2.3.2. Branched PNIPAM Synthesis

The architecture of a polymer significantly influences the properties of synthesized smart materials. Compared to their linear counterparts, branching in polymers can lead to distinct behaviors due to reduced chain entanglement and altered hydrodynamic volume [24,25,26]. In this context, a branched PNIPAM was grafted onto the substrate material to explore its water vapor desorption performance, contrasting it with the previously described linear PNIPAM.

The first stage Involved Immobilizing the ATRP-initiator, following the same approach as grafting linear PNIPAM. The ATRP method enabled PNIPAM growth from the substrate′s hydrophilic surface in the second stage. For synthesizing branched PNIPAM, the polymerization process encompassed three essential steps: initial grafted PNIPAM polymerization, incorporation of EBMA to introduce branching points and subsequent PNIPAM polymerization from EBMA. The procedure commenced with introducing a methanol and Milli-Q water mixture (30:10 *v*/*v*) into a round-bottom flask fitted with a magnetic stirrer. Subsequently, the flask was sealed and connected to an active nitrogen gas line, which was immersed in an ultrasonic bath for 30 min to establish an inert atmosphere. The modified BiB–cotton substrate, accompanied by a catalytic amount of CuBr and NIPAM, was introduced into a Schlenk flask. Following this, a methanol/water solution and PMDETA were added to the inert flask, and the solution was cooled using liquid nitrogen until complete freezing was achieved. Successive freeze–vacuum–nitrogen–thaw cycles further degassed the solution. The flask was then attached to a vacuum line and submerged in liquid nitrogen until complete solvent freezing. The vacuum was initiated for 2–3 min, and the flask was removed from the liquid nitrogen to enable solvent thawing, facilitating the escape of trapped gas bubbles into the flask′s headspace. This cycle was repeated three times. Nitrogen was introduced into the flask and then sealed with a balloon to establish positive pressure. The ensuing reaction was conducted at 40 °C with magnetic stirring for an hour. Following this, EBMA was added to the solution, and the reaction continued at 40 °C with magnetic stirring for 48 h. Subsequently, the PNIPAM substrate product underwent a thorough triple rinsing sequence with ethanol, acetone, and water. Finally, the product was dried at 50 °C under vacuum conditions for 24 h.

#### 2.3.3. Extended Purification Procedure

The PNIPAM substrate smart material was designed for interaction with the human breathing cycle, aiming to humidify inspired air using moisture recovered from expired air. As a result, prior to initiating the humidification experiments, it was crucial to thoroughly purify the material and eliminate any traces of monomer, catalyst, and solvent. Initially, the PNIPAM substrate underwent Soxhlet extraction with methanol under reflux for an hour to remove residual non-reacted monomer, remaining catalyst complexes, and any polymer absorbed onto the surface. Following the extraction, the PNIPAM substrate was dried at 50 °C under vacuum for 24 h and subsequently stored in a 50 °C incubator to ensure complete dryness prior to commencing the subsequent series of experiments.

#### 2.3.4. Synthesis of PNIPAM with Different Mw (5 kDa, 15 kDa, 50 kDa)

To calculate the quantity of NIPAM required to polymerization the PNIPAM from the BiB substrate with different Mw (5 kDa or 5000 g/mol, 15 kDa or 15,000 g/mol, and 50 kDa or 50,000 g/mol), considering that one PNIPAM chain (1 mol) use one BiB molecule attached to the substrate (1 mol) to grow on the substrate. Hence, the quantity of NIPAM required to be grafted in both substrates is determined from

$$g\;\mathrm{BiB}\;\mathrm{reacted}\cdot \frac{1\;\mathrm{mol}\;\mathrm{BiB}}{229.9\;g}\cdot \;\frac{1\;\mathrm{mol}\;\mathrm{PNIPAM}}{1\;\mathrm{mol}\;\mathrm{BiB}}\cdot \;\frac{M\;g\;\mathrm{PNIPAM}}{1\;\mathrm{mol}\;\mathrm{PNIPAM}}\approx g\;\mathrm{NIPAM}$$

where M = molecular weight of the PNIPAM.

### 2.4. Smart Material Characterization

Various characterization methods were employed to examine different aspects of the polymerization process, including optimizing the PNIPAM synthesis routine, assessing its thermal transition performance, and determining the final Mw.

#### 2.4.1. Proton Nuclear Magnetic Resonance (^1^H NMR)

Nuclear magnetic resonance spectroscopy was employed to monitor the polymerization process and monomer conversion over time. This technique was chosen to identify the carbon–hydrogen framework within an organic compound. The ^1^H NMR measurements were conducted using a Bruker AscendTM 400 MHz spectrometer. The proton chemical shifts were recorded in ppm using tetramethylsilane (TMS) as a reference. Each measurement was averaged from 16 scans. Methanol-d4 was employed as a solvent to assess the monomer conversion over time, and DMF served as an internal reference. Samples from the reaction mixture were collected at 0, 1, 8, 24, and 48 h from the start of the reaction and then promptly injected into sealed NMR tubes for ^1^H NMR experiments. The obtained NMR spectra were processed using Bruker′s software (TopSpin v3.5) and exported for analysis using MestReNova v12.0.2 software.

#### 2.4.2. Fourier Transform Infrared (FTIR) Spectroscopy

Fourier transform infrared spectroscopy is a technique employed to generate an infrared spectrum reflecting the absorption or emission of a solid, liquid, or gas sample. This analytical method finds extensive use, particularly for identifying polymers and organic compounds. Through FTIR spectroscopy, an ordered spectrum was produced, facilitating the identification or quantification of the analyzed sample due to distinct molecular infrared fingerprints. In this study, FTIR spectroscopy was employed to validate the reaction′s success by comparing the spectra of the unmodified substrate with those of the BiB substrate and the PNIPAM substrate.

#### 2.4.3. Energy Dispersive X-ray Spectroscopy (EDS)

Energy dispersive X-ray spectroscopy is a chemical analysis technique with scanning electron microscopy (SEM). This study employed EDS data to determine the chemical composition of the unmodified substrate, the BiB substrate, and the synthesized PNIPAM substrate. The SU-70 Analytical Field Emission Scanning Electron Microscope (Hitachi, Tokyo, Japan) was employed for EDS spectra, using NSS software for X-Ray microanalysis v3.0 (ThermoFisher Scientific, Waltham, MA, USA). An acceleration voltage of 15 kV and a magnification of 2000× were set. Finally, the recorded spectrum of X-ray energy plotted against counts was analyzed to deduce the elemental composition of the scanned sample.

#### 2.4.4. Simultaneous Thermal Analysis

STA is a technique used to simultaneously analyze differential scanning calorimetry (DSC) and thermal gravimetric analysis (TGA) on a sample under identical test conditions. DSC analysis calculates the heat required to raise the sample temperature compared to a reference over time. This hermos-analytical method primarily analyzes the thermal transitions of polymer materials. To maintain the same temperature when the sample undergoes a physical transformation, heat will need to flow into the sample compared to the reference, depending on the process (exothermic or endothermic). The output of a DSC experiment is the heat flux versus temperature or time [27,28]. This method was used to calculate the LCST and degradation temperature of the synthesized PNIPAM substrates.

TGA was used to obtain measurements of the physiochemical behaviors of materials, such as absorption and desorption, phase transitions, or chemical oxidation and reduction. The output of a TGA experiment is a plot of mass or percentage of initial mass versus temperature or time, which can be used to analyze decomposition patterns in polymers [27,28]. The quantity of water vapor released from the smart material was determined by calculating the mass reduction corresponding to the released amount of water vapor.

STA experiments for the PNIPAM substrate samples were conducted using a Jupiter STA 449 F5 (Netzsch, Selb, Germany), and both the analysis and experiment setup were carried out with Netzsch Proteus Software v6.1. All samples were kept at 100% relative humidity (RH) and room temperature for four hours to ensure saturation and maximum water vapor absorption. A temperature profile with a 15 °C/min heating rate and under argon gas flow at 20 mL/min, ranging from 20 °C to 200 °C, was used to create the baseline. The weighted sample was placed inside an aluminum pan with a punctured aluminum lid to allow water vapor to leave the aluminum pan as the temperature increased.

### 2.5. Statistical Analysis

For statistical analysis, one-way and two-way analyses of variance (ANOVA) were conducted using GraphPad Prism software version 6.07 for Windows (GraphPad et al., San Diego, CA, USA). The results are reported as the mean ± standard deviation (SD).

## 3. Results and Discussion

The goal was to create an intelligent material capable of efficiently absorbing and releasing water vapor during a breath cycle of 4–6 s. This was achieved by grafting linear and branched PNIPAM brushes onto PVA and cotton substrates using an ATRP procedure. The resulting smart material underwent characterization, examining the success of the reaction, morphology, chain length or Mw, and water vapor desorption performance. A comparison was made between PNIPAM grafted on PVA and cotton substrates, followed by comparing the performance between linear and branched PNIPAM. Additionally, PNIPAM with different Mw (5, 15, and 50 kDa) was assessed to optimize the smart material’s performance for humidifying inspired gas using recovered moisture from the expired flow.

### 3.1. Analysis of Substrate Material: PVA vs. Cotton

**Figure 2:**
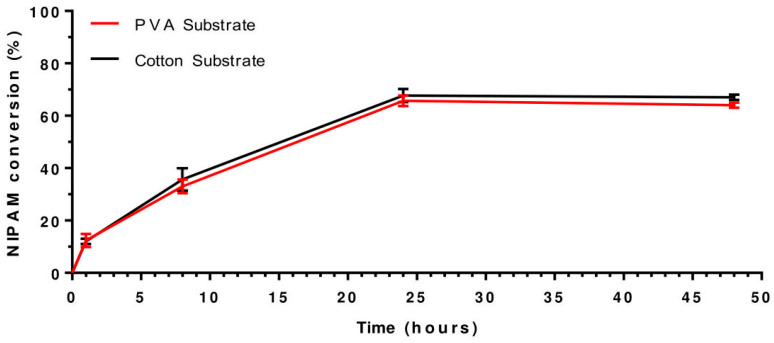
The NIPAM conversion (%) over time for PNIPAM synthesis of PVA (red line) and cotton (black line).

To analyze the optimum substance for the smart material, we initially investigated the success of the first reaction and the presence of BiB using EDS. Table 1 (Analysis 2) summarizes the results for three PVA and three cotton samples, showing the amount of BiB reacted based on the weight (%) of Br from EDS data. Values from the weight difference method and EDS data align with no significant difference. The amount of BiB reacted on the PVA substrate was significantly lower (*p* < 0.0001) compared to the quantity on the cotton fabric due to the two substrates’ contrasting physical structures. EDS analysis was also performed to assess the success of PNIPAM synthesis on PVA and cotton fabric. The critical molecular distinction between the BiB substrates and PNIPAM is the presence of nitrogen (N) (Table 2: Analysis 3). The quantity of PNIPAM on the substrate was determined by comparing the sample weight before and after the grafting reaction. The weight difference indicates the amount of PNIPAM synthesized. In Table 1 (Analysis 4), the third column in the table shows the amount of PNIPAM reacted, calculated based on the weight (%), and the fourth column represents the amount of polymer reacted, derived from the NIPAM conversion (%) determined by NMR spectra evolution over time. The calculated amounts of PNIPAM from the weight difference method, EDS data, and NMR results have no significant difference. The PNIPAM reaction on the PVA substrate shows significantly lower results (*p* < 0.0001) than the cotton fabric in all three methods. This difference can be attributed to the lower amount of BiB reacted on the PVA substrate, resulting in fewer grafting points available for NIPAM grafting (Appendix A). Figure 2 illustrates the NIPAM conversion (%) over time during PNIPAM synthesis on both PVA and cotton substrates.

**Figure 3:**
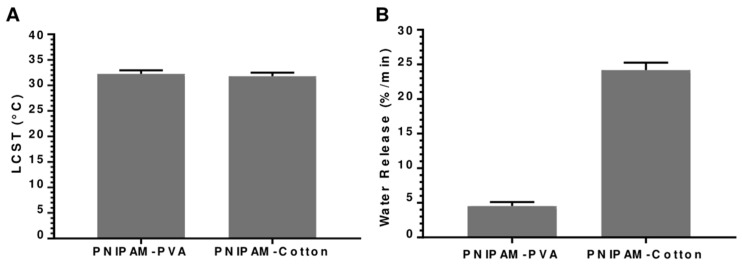
STA results. (**A**) LSCT of PNIPAM-PVA and PNIPAM–cotton substrates determined from the DSC curve. (**B**) Water vapor release (%/min) of PNIPAM-PVA and PNIPAM–cotton substrates determined from the TGA curve.

To effectively compare the performance of both substrates, it was essential to determine the theoretical chain length or Mw of the grafted PNIPAM. Since one PNIPAM chain grows from one BiB molecule attached to the substrate, the amounts of PNIPAM and BiB reacted on each substrate were already established. Assuming all chains had uniform lengths, the theoretical Mw of the PNIPAM grafted from the PVA substrate was 9713.27 Da or g/mol, while the theoretical Mw of the PNIPAM grafted from the cotton substrate was 10,099.59 Da or g/mol. Consequently, we could assess the water vapor desorption rate for both samples, as their chain lengths fall within the same order of magnitude. Linear PNIPAM grafted on PVA, and cotton substrates were compared and analyzed to investigate the performance of the smart material using STA analysis. While the LCST determined In both PNIPAM substrates was around 32 °C as theoretically expected, the PNIPAM-PVA samples demonstrated a significantly lower water vapor release rate than the PNIPAM–cotton samples. This observation can be attributed to the fact that the number of PNIPAM chains grown from the PVA substrate was seven times less than that from the cotton substrate. Therefore, the cotton fabric was chosen for further investigation (Figure 3).

### 3.2. Analysis of Polymer Structure: Linear vs. Branched

To analyze the optimum polymer structure, we initially investigated the success of the first reaction and the presence of BiB using EDS. The weight percentage of C, O, and Br were determined for the six samples at different surface points as 49.390 ± 1.491, 43.958 ± 1.259, and 5.432 ± 0.721, respectively. The weight difference representing the amount of reacted BiB was 0.126 ± 0.014. However, the amount of BiB calculated from the weight (%) of Br from the EDS data was 0.138 ± 0.008. Thus, the weight difference method and EDS data aligned with no significant difference.

The weight percentages of C, O, and N were analyzed for the three branched and three linear samples at various surface points and summarized in Table 1 (Analysis 5). The quantity of PNIPAM on the substrate was determined by comparing the sample weight before and after the grafting reaction and summarized in Table 2 (Analysis 6). The amount of PNIPAM reacted, expressed as a weight percentage, was determined using EDS, where one mole of N corresponds to one mole of PNIPAM (Mw of 16 g/mol). The calculated PNIPAM amount from the weight difference method and EDS data closely matched, with no significant difference observed. The PNIPAM reaction followed by the branched synthesis method yielded significantly lower results (*p* < 0.001) than the linear synthesis method. This difference can be attributed to the probability of side reactions occurring, resulting in synthesizing a side PNIPAM product with the EBMA rather than growing branches from the substrate. Both theoretical branched and linear structures were analyzed with STA to compare their water desorption performance and determine the optimal structure.

**Figure 4:**
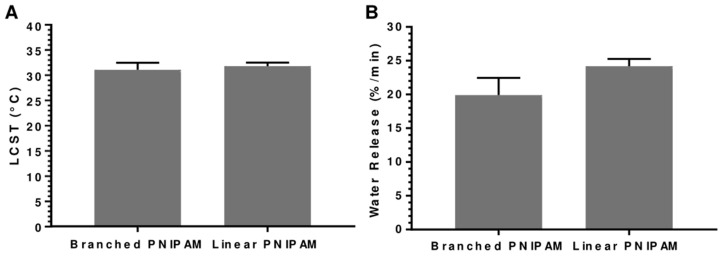
STA results. (**A**) LSCT of branched and linear PNIPAM–cotton substrates determined from the DSC curve. (**B**) Water vapor release (%/min) of branched and linear PNIPAM–cotton substrates determined from the TGA curve [30].

The linear PNIPAM and branched PNIPAM were compared and analyzed to investigate the performance of the smart material. While the LCST determined in both PNIPAM structures was around 32 °C, as theoretically expected, the branched PNIPAM samples demonstrated a significantly lower water vapor release rate than the linear PNIPAM samples. This observation can be attributed to the lower number of polymer chains grafted on the substrate in the branched PNIPAM samples compared to the linear PNIPAM samples. Furthermore, the synthesized branched PNIPAM can potentially impede the conformational change from the linear to coiled structure, reducing the rate of water vapor release [25,29]. Therefore, the linear PNIPAM synthesized in a cotton fabric was chosen for further investigation (Figure 4).

### 3.3. Analysis of Polymer Molecular Weight: 5, 15, 50 kDa

**Figure 5:**
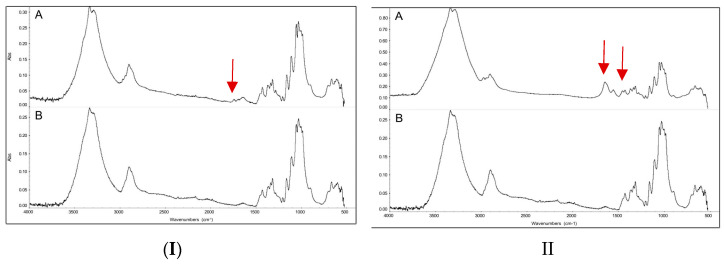
FTIR spectra obtained in absorbance mode. (**I**-**A**) Spectra of BiB–cotton fabric; (**I**-**B**) bare cotton fabric; (**II**-**A**) spectra of PNIPAM–cotton fabric; (**II**-**B**) bare cotton fabric.

According to the substrate and polymer structure analyses, the most favorable characteristics for water vapor desorption were exhibited by the linearly grafted PNIPAM onto the cotton substrate. The primary focus of this analysis was to optimize the Mw of PNIPAM on the substrate. To determine the optimum Mw, the success of the first reaction, which was the immobilization of BiB (ATRP-initiator) on the substrate material, was initially investigated by employing FTIR analysis in absorbance mode and comparing the spectra from the bare and BiB-modified cotton fabric. The BiB–cotton samples displayed a single peak at 1740 cm^−1^, corresponding to the stretching vibration of the ester carbonyl group that connected the BiB to the cotton fabric. This peak did not appear in the spectra of the bare cotton fabric (Figure 5I). 

In addition to analyzing the FTIR spectra, the presence of BiB on the cotton was examined using EDS. The weight percentages of C, O, and N were determined for all nine samples at various surface points as 49.632 ± 1.431, 44.066 ± 1.135, and 5.567 ± 0.647, respectively. The weight difference representing the amount of reacted BiB was 0.128 ± 0.044. The amount of reacted BiB was calculated using the weight (%) of Br obtained from the EDS data and was 0.157 ± 0.008. The calculated BiB amount from both the weight difference method and the EDS data closely agree with each other.

**Figure 6:**
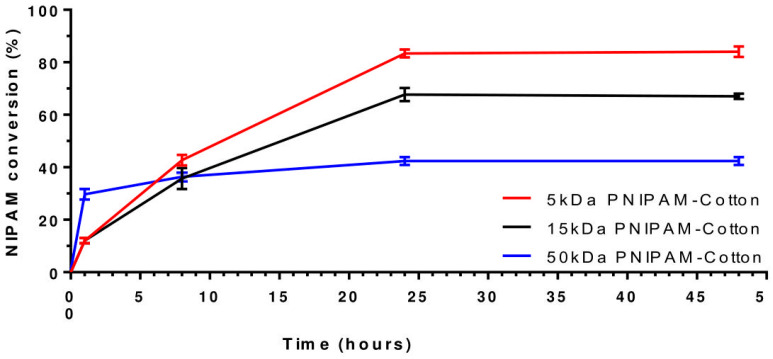
The percentages of NIPAM conversion over time 5 kDa (red line), 15 kDa (black line), and 50 kDa (blue line).

The necessary quantity of NIPAM for grafting onto both substrates was determined based on the target final Mw of PNIPAM. However, the critical point for the calculation was that one PNIPAM chain (1 mol) grows from one BiB molecule attached to the substrate (1 mol). The NIPAM conversion at different Mw was investigated through NMR analysis. Samples were taken from the reacting mixture at various time points during the polymerization process (0, 1, 8, 24, and 48 h). The NMR spectra obtained were analyzed by comparing them to reference signals. The 0 h time duration corresponds to the 0% conversion of the monomer (Figure 6). FTIR in the absorbance mode was employed to assess the success of the PNIPAM synthesis. Figure 5II shows the FTIR spectra for both the bare cotton and the PNIPAM–cotton samples. The PNIPAM–cotton samples exhibited two peaks for the stretching vibrations of amide and carbonyl groups of PNIPAM at 1650 cm^−1^ and 1540 cm^−1^, respectively, which were not present in the bare cotton spectra. The absorbed water was observed at a vibration band of 1635 cm^−1^.

EDS analysis was performed to assess the success of PNIPAM synthesis on cotton fabric. The critical molecular distinction between the BiB substrates and the PNIPAM was the presence of N. The weight percentages of C, O, and N were determined at different surface points for three samples at each Mw (5 kDa, 15 kDa, and 50 kDa) and are presented in Table 1 (Analysis 7).

In Table 2 (Analysis 8), the third and fourth columns show the amount of PNIPAM reacted from EDS and NMR, respectively. The results show no significant difference. The monomer conversion displayed a logarithmic increase and eventually stabilized after 24 h. The 5 kDa and 15 kDa curves exhibit a similar pattern, with the 15 kDa sample reaching monomer conversions of 67% and 84%, respectively. In contrast, the 50 kDa curve shows a rapid initial monomer conversion within the first hour, but its stabilization was slower, reaching only 42% conversion. This difference can be attributed to the significantly higher monomer concentration required for producing a high Mw polymer, which may hinder the monomer′s free movement or lead to saturation.

**Figure 7:**
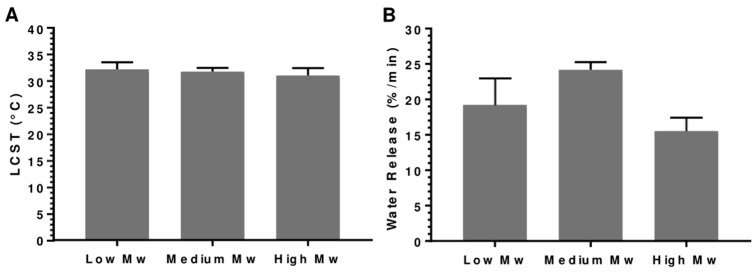
STA results. (**A**) LSCT of low, medium, and high Mw PNIPAM–cotton samples determined from the DSC curve. (**B**) Water vapor release (%/min) of low, medium, and high PNIPAM–cotton samples determined from the TGA curve [30].

Different Mw (5, 15, and 50 kDa) of linear PNIPAM grafted onto a cotton fabric substrate were compared and analyzed to investigate the performance of the smart material. While the LCST determined in both the PNIPAM structures was around 32 °C, as theoretically expected, both low and high Mw samples demonstrated a significantly lower water vapor release rate than the medium Mw PNIPAM samples. This observation can be attributed to the number of PNIPAM chains grafted onto the substrate. Specifically, the low Mw samples have a lower number of grafted PNIPAM chains, resulting in the absorption of fewer water vapor molecules. In comparison, the high Mw samples absorb more molecules, leading to slower release than the medium Mw samples [31,32]. Therefore, medium Mw (between 7 and 20 kDa) was chosen as the optimum Mw for the smart material, Figure 7.

### 3.4. Assessment of the Optimized PNIPAM Smart Material

This study aimed to synthesize a smart material for humidifying inhaled gas using reclaimed moisture from exhaled airflow while achieving a predetermined humidity level. The PNIPAM smart polymer with thermo-responsive properties was optimized to maximize its capacity to absorb water vapor and release it quickly.

Two materials, PVA and organic cotton fabric, were examined for their suitability in achieving optimal PNIPAM grafting efficiency. The results reveal a notable difference in the amount of BiB (a reactive agent) that reacted with these substrates. Specifically, the quantity of BiB reacted on cotton fabric was significantly higher than on the PVA substrate. This variation can be attributed to the distinct physical structures of these materials. The cotton fabric is porous, allowing for easier access of the BiB reagent to interact with the OH groups. At the same time, the sponge-like structure of PVA might have impeded BiB interaction with the available OH groups.

Furthermore, the quantity of PNIPAM grafted onto the PVA substrate was considerably lower than that on the cotton fabric across all three methods. This outcome was anticipated due to the lower amount of reacted BiB on PVA, resulting in fewer sites for grafting NIPAM. Notably, the LCST determined that both PNIPAM substrates exhibited no significant divergence, aligning with the theoretical expectation at approximately 32 °C. However, there was a significant distinction in the rate of water vapor release from the PNIPAM-PVA samples compared to the PNIPAM–cotton samples. Despite the comparable Mw of grafted PNIPAM chains (around 10 kDa) in both substrates, the number of polymer chains originating from the PVA substrate was significantly lower, approximately one-seventh of that from the cotton substrate. As a result, the cotton fabric substrate was deemed more suitable for subsequent experiments due to its higher count of BiB sites, which were available for further reactions.

The observed disparity in the release rate of water vapor between the PNIPAM-PVA and PNIPAM–cotton samples can be traced back to the unique physical attributes of these substrates, which significantly influenced the growth of PNIPAM chains. The cotton substrate, characterized by its fabric-like structure, provided ample space between its fibers. This spatial arrangement facilitated an extensive interaction between the BiB and the OH groups on the cotton, resulting in a higher number of available sites for NIPAM grafting. Consequently, an increased number of PNIPAM chains formed on the cotton substrate. This abundance of PNIPAM chains was pivotal for enhancing the release of water vapor since PNIPAM has the capacity to efficiently uptake and release water vapor in response to temperature fluctuations. Conversely, the PVA substrate consists of a sponge-like structure, which, regrettably, can pose a barrier to BiB access and hinder its interaction with the OH groups deep within the interior of the material. This obstacle limited the number of available sites for NIPAM growth on the PVA substrate, resulting in a reduced number of PNIPAM chains. Consequently, the release rate of water vapor was diminished, primarily due to the fewer polymer chains available to capture and release moisture from the surrounding environment.

Subsequently, the cotton substrate was used to synthesize both linear and branched PNIPAM to compare their respective rates of water desorption. The outcomes reveal a significant discrepancy: the quantity of PNIPAM that underwent reaction using the branched synthesis method was markedly lower compared to the amount engaged by the linear synthesis approach. The side reactions led to the potential formation of unintended byproducts, such as EBMA generating a side PNIPAM product rather than the desired growth of branches from the substrate. However, investigating these potential side reactions was not pursued further in this study, as it diverged from the primary research objective. The STA analysis indicated no significant difference in the LCST between the two PNIPAM structures, aligning with the theoretical expectation of around 32 °C. However, a notable observation was exhibited in the water vapor release rate from the branched PNIPAM samples in contrast to the linear PNIPAM samples. This disparity can be attributed to the lower extent of polymer grafting onto the substrate in the case of branched PNIPAM, which reduced water vapor absorption capacity. Furthermore, it is plausible that the synthesized PNIPAM branches could hinder the transition from a linear to a coiled conformation, resulting in a slower water release [25,29,31,32]. In summary, the pivotal factor behind the variation in water vapor release rates between the linear and branched polymer structures was the number of PNIPAM (linear and branched) chains available for capturing and releasing water vapor, along with the ability to change its structure in response to temperature fluctuations. These factors influenced its capacity to interact with and release moisture as the temperature changed.

Finally, linear PNIPAM was synthesized on the cotton substrate with Mw (below 7 kDa, between 7 and 20 kDa, and above 20 kDa). The STA analysis demonstrated no significant distinction in the LCST among the three PNIPAM samples with varying Mw, consistently hovering around 32 °C, in line with the theoretical expectations. However, a substantial contrast was observed in the water vapor release rate between the low- and high-molecular-weight PNIPAM samples compared to the medium-molecular Mw PNIPAM samples. This phenomenon can be rationalized by the fact that the low Mw samples lacked a sufficient amount of grafted PNIPAM, resulting in diminished absorption of water vapor molecules compared to the medium MW samples. Conversely, the high Mw PNIPAM samples exhibited the highest ability to absorb water vapor molecules, but their release rate was comparatively slower than that of the medium MW samples [25,29,31,32]. In summary, the pivotal factor behind the variation in water vapor release rates between different Mw polymers was the number of PNIPAM chains available for capturing and releasing water vapor and the ability to release captured water vapor upon temperature fluctuations efficiently. Based on the above analysis, cotton fabric was chosen as the substrate, and a linear PNIPAM structure with a medium Mw (between 7 and 20 kDa) was selected for the final optimization. Therefore, the medium Mw (between 7 and 20 kDa) PNIPAM–cotton fabric substrate was chosen for the application. The optimized thermo-responsive material exhibited a water vapor release rate of 24.2 ± 1.054% per minute at the material′s LCST of 32 °C. These results indicate that there were no significant differences among the three samples that were analyzed. Thus, the results are consistent and can be repeated reliably.

When assessing the material′s biocompatibility, it is essential to separately evaluate the biocompatibility of both the substrate and the polymer. As one of the most abundant biopolymers on Earth, cotton inherently possesses remarkable biocompatibility properties. These natural characteristics make it a highly suitable substrate for biomedical and biotechnological applications. The biocompatibility of PNIPAM polymer has been unequivocally established through a series of robust studies [1,33,34,35,36,37,38]. Matsuda and colleagues illustrated this biocompatibility by showing the in situ formation of a PNIPAAm-gelatin gel within the subcutaneous tissues of rats, highlighting its compatibility with biological environments [35]. A recent investigation further fortified this claim by successfully conducting intravitreal injections of pure PNIPAM into rabbit eyes, reaffirming its suitability for biological applications [1]. These findings are complemented by diverse approaches taken in other studies. Das et al. engineered covalently crosslinked PNIPAAm hydrogels designed for oral drug delivery using dextrin and NIPAm as components, while Wei et al. introduced salecan, a natural hydrophilic polysaccharide, into PNIPAM hydrogel networks, enhancing both mechanical strength and biocompatibility [36,37]. The incorporation of modifications, such as grafting PNIPAM onto graphene sheets and synthesizing PNIPAM copolymers, further attests to the material′s suitability for various biomedical applications. Together, these findings solidify the well-documented biocompatibility of PNIPAM polymer, making it a promising candidate for various biological and medical uses. Furthermore, a recent study reported by Malihe et al., who employed analogous chemicals and methodologies to synthesize a PNIPAM polymer, conducted in vitro biological analyses, affirming the biocompatibility of this similar PNIPAM polymer structure [38]. In conjunction with the previously mentioned evidence, these findings strongly support our claim regarding the material′s biocompatibility.

## 4. Conclusions

This research pioneers using thermo-responsive PNIPAM as the primary substance for the humidification process in lung support devices, offering a cost-effective and environmentally friendly approach. The use of this polymer prompts the consideration of other smart polymers that could potentially replace bulky, high-energy-consuming humidification equipment in biomedicine. By optimizing the response to external stimuli and the desired performance, this innovation opens doors to more efficient and sustainable biomedical solutions. The thermo-responsive properties of the PNIPAM were optimized to absorb and release moisture at the LCST efficiently. The results demonstrate a minimum water vapor release rate of 24.2 ± 1.054% at an LCST of 32 °C. Significant characteristics successfully achieved the research objective of utilizing recovered moisture from exhaled air to humidify inhaled air.

## 5. Patents

WO 2019/093910 Al.

## Figures and Tables

**Table 1:** The quantity of BiB/PNIPAM present in the substrates from different methods for different samples in different analyses. All the studies were triplicated, so the number of samples for each analysis was *n* = 3.

Condition	Calculated BiB/PNIPAM from Different Methods		
	Weight Difference:	EDS Data:	NMR Data:
Analysis 2: Presence of BiB on the substrate			
PVA	0.034 ± 0.015	0.028 ± 0.008	N/A
Cotton	0.158 ± 0.054	0.173 ± 0.026	N/A
Analysis 4: PNIPAM synthesis on the substrate			
PVA	1.015 ± 0.125	1.114 ± 0.183	1.183 ± 0.182
Cotton	6.529 ± 0.107	7.110 ± 0.421	6.941 ± 0.851
Analysis 6: Analysis of polymer structure			
Branched	3.815 ± 0.125	4.014 ± 0.583	N/A
Linear	6.529 ± 0.107	7.110 ± 0.421	N/A
Analysis 8: Analysis of polymer MW.			
5 kDa	1.931 ± 0.125	2.512 ± 1.583	2.329 ± 1.982
15 kDa	6.529 ± 0.107	7.110 ± 1.421	6.941 ± 1.851
50 kDa	10.627 ± 0.118	9.927 ± 1.219	11.784 ± 1.957

**Table 2:** The EDS data display the average weight percentages (%) of C, O, and Br of different samples (BiB on cotton and PVA, PNIPAM on cotton and PVA, linear and branched PNIPAM on cotton, linear PNIPAM with different MW on cotton). All the analyses were triplicated, so the number of samples for each analysis was *n* = 3.

Substrate/PNIPAM Structure/PNIPAM MW	Weight (%) of C	Weight (%) of O	Weight (%) of Br
Analysis 1: Presence of BiB on the substrate			
PVA	48.517 ± 1.231	44.700 ± 0.550	3.817 ± 0.685
Cotton	49.533 ± 1.978	43.613 ± 1.636	5.920 ± 0.387
Analysis 3: PNIPAM synthesis on the substrate			
PVA	48.677 ± 0.956	37.803 ± 1.488	10.873 ± 0.646
Cotton	48.860 ± 1.010	37.231 ± 1.328	11.630 ± 1.324
Analysis 5: Analysis of polymer structure			
Branched	48.657 ± 0.956	41.060 ± 1.488	10.283 ± 0.646
Linear	48.860 ± 1.010	37.231 ± 1.328	11.630 ± 1.324
Analysis 7: Analysis of polymer MW			
5 kDa	47.783 ± 2.252	37.230 ± 1.328	10.553 ± 1.072
15 kDa	48.860 ± 1.010	37.231 ± 1.328	11.630 ± 1.324

## Data Availability

Contact the corresponding author.

## Appendix A

### Analysis of Substrate Material: PVA vs. Cotton

As the first step, the number of OH groups available was quantified, and the excess amount of BiB reagent needed to succeed in the substrate surface modification reaction was determined. One PVA molecule has only one OH group, so one mole of PVA corresponds to one mole of OH and one mole of BiB since one BiB molecule reacts with one OH group. However, an excess of three-times was used in the reaction.

BiB (ATRP-initiator) immobilized on the substrate material was investigated by analyzing the samples using EDS. It was aimed to detect the molecular difference between the substrates and the BiB–cotton, which was the amount of bromine (Br). The weight percentages of carbon (C), oxygen (O), and Br were determined at various surface points for three PVA and three cotton samples. The results are presented in Table 1 (Analysis 1). The amount of BiB on the substrate was determined by comparing the sample weights before and after the BiB reaction. The resulting weight difference indicates the quantity of BiB reacted. Table 2 (Analysis 2) summarizes the results for the three PVA and three cotton samples, showing the amount of BiB reacted based on the weight (%) of Br from EDS data. One mole of Br (Mw 79.9 g/mol) corresponds to one mole of BiB. Values from the weight difference method and EDS data align with no significant difference. The amount of BiB reacted on the PVA substrate was significantly lower (*p* < 0.0001) compared to the quantity reacted on the cotton fabric owing to the contrasting physical structures of the two substrates. The cotton fabric′s fibers had more space between them, allowing the BiB easier access to react with the OH groups. In contrast, the PVA substrate′s sponge-like structure might have obstructed BiB entry, hindering its reaction with the OH groups within its interior.

To determine the amount of NIPAM required to synthesize PNIPAM from the BiB substrate with a Mw of 15 kDa (15000 g/mol), it is essential to consider that one PNIPAM chain (1 mol) grows from one BiB molecule attached to the substrate (1 mol). Thus, the required amount of NIPAM to be grafted on both substrates could be calculated. The NMR spectra were acquired, and the NIPAM signals were integrated relative to the DMF signals. The sample analyzed at time 0 served as the reference for 0% monomer conversion. Figure 2 illustrates the NIPAM conversion (%) over time during the PNIPAM synthesis on both PVA and cotton substrates. The monomer conversion progressively increased in a logarithmic manner and reached stability after 24 h, resulting in a 65% conversion from the PVA substrate and a 67% conversion from the cotton substrate. Notably, the two curves are similar.

EDS analysis was performed to assess the success of PNIPAM synthesis on PVA and cotton fabric. The critical molecular distinction between the BiB substrates and PNIPAM was the presence of nitrogen (N). The weight percentages of C, O, and N were determined at different surface points for three PVA and three cotton samples. The summarized outcomes for each substrate material are presented in Table 1 (Analysis 3).

The quantity of PNIPAM on the substrate was determined by comparing the sample weight before and after the grafting reaction. The weight difference indicates the amount of PNIPAM synthesized. In Table 2 (Analysis 4), the third column in the table shows the amount of PNIPAM reacted, calculated based on the weight (%) of N determined from the EDS data, where one mole of N (Mw of 16 g/mol) corresponds to one mole of PNIPAM. Furthermore, the fourth column represents the amount of polymer reacted, derived from the NIPAM conversion (%) determined by the NMR spectra evolution over time. The calculated amounts of PNIPAM from the weight difference method, EDS data, and NMR results have no significant difference. The PNIPAM reaction on the PVA substrate shows significantly lower results (*p* < 0.0001) than the cotton fabric in all three methods. This difference can be attributed to the lower amount of BiB reacted on the PVA substrate, resulting in fewer grafting points available for NIPAM grafting.

To effectively compare the performance of both substrates, it is essential to determine the theoretical chain length or Mw of the grafted PNIPAM. Since one PNIPAM chain grows from one BiB molecule attached to the substrate, the amounts of PNIPAM and BiB reacted on each substrate are already established. Assuming all chains have uniform lengths, the theoretical Mw of the PNIPAM grafted from the PVA substrate is 9713.27 Da or g/mol, while the theoretical Mw of the PNIPAM grafted from the cotton substrate is 10099.59 Da or g/mol. Consequently, both samples can assess the water vapor desorption rate, as their chain lengths fall within the same order of magnitude.

Linear PNIPAM grafted on PVA, and cotton substrates were compared and analyzed to investigate the performance of the smart material using STA analysis. While the LCST determined in both PNIPAM substrates was around 32 °C as theoretically expected, the PNIPAM-PVA samples demonstrated a significantly lower water vapor release rate than the PNIPAM–cotton samples. This observation can be attributed to the fact that the number of PNIPAM chains grown from the PVA substrate was seven times less than from the cotton substrate. Therefore, the cotton fabric was chosen for further investigation (Figure 3).
